# Treating tuberculosis with high doses of anti-TB drugs: mechanisms and outcomes

**DOI:** 10.1186/s12941-017-0239-4

**Published:** 2017-10-03

**Authors:** Yuhui Xu, Jianan Wu, Sha Liao, Zhaogang Sun

**Affiliations:** 1 Institute of Chinese Materia Medica, China Academy of Chinese Medical Science, Beijing, 100700 China; 2 National Tuberculosis Clinical Laboratory, Beijing Chest Hospital, Capital Medical University, 9 Beiguan Street, Tongzhou District, Beijing, 101149 China; 3 Beijing Key Laboratory in Drug Resistant Tuberculosis Research, Beijing Tuberculosis & Thoracic Tumor Research Institute, Beijing, 101149 China

**Keywords:** Tuberculosis, Treatment, Anti-TB drugs, High dosage

## Abstract

Tuberculosis (TB) is considered as one of the most serious threats to public health in many parts of the world. The threat is even more severe in the developing countries where there is a lack of advanced medical amenities and contemporary anti-TB drugs. In such situations, dosage optimization of existing medication regimens seems to be the only viable option. Therapeutic drug monitoring study results suggest that high-dose treatment regimens can compensate the low serum concentration of anti-TB drugs and shorten the therapy duration. The article presents a critical review on the possible changes that occur in the host and the pathogen upon the administration of standard and high-dose regimens. Some of the most common factors that are responsible for low anti-TB drug concentrations in the serum are differences in hosts’ body weight, metabolic processing of the drug, malabsorption and/or drug–drug interaction. Furthermore, failure to reach the cavitary pulmonary and extrapulmonary tissues also contributes to the therapeutic inefficiency of the drugs. In such conditions, administration of higher doses can help in compensating the pathogenic outcomes of enhancement of the pathogen’s physical barriers, efflux pumps and genetic mutations. The present article also presents a summary of the recorded treatment outcomes of clinical trials that were conducted to test the efficacy of administration of high dose of anti-tuberculosis drugs. This review will help physicians across the globe to understand the underlying pathophysiological changes (including side effects) that dictate the clinical outcomes in patients administered with standard and/or high dose anti-TB drugs.

## Background

Tuberculosis is a highly contagious disease caused due to *Mycobacterium tuberculosis* infection. Though the methods of treatment of the disease have been standardized since long, many people, especially in the developing countries, still succumb to it. According to recent statistical figures published by the World Health Organization (WHO) [1], 10.4 million new cases of TB were reported worldwide in the year 2015 alone. It was also observed that while 1.4 million died of the disease in the same year, an estimated 480,000 were diagnosed with multidrug-resistant TB [MDR-TB, defined as resistant to at least isoniazid (INH) and rifampin (RFP)] and an additional 100,000 with rifampicin-resistant TB (RR-TB). Since the standard treatment regimens are ineffective in the treatment of such patients, they were compelled to undertake the MDR-TB specific treatment. Hence, it is proposed that the advent and worldwide distribution of new anti-TB drugs (MDR-TB drugs) is indispensable for winning the war against TB on a global level. Interestingly, it was observed that over 95% of the total number of TB associated fatality cases recorded in 2015 occurred in low- and middle-income countries. This points towards the need of improving the standards of medical care and accessibility to traditional as well as contemporary (MDR) anti-TB drugs, which can be achieved by lowering the prices and maintaining consistent supplies. However, the process of development of new (MDR) anti-TB drugs that has improved efficacy and safety is still under experimentation. The only option to control the situation presently is to devise new methods that can help in deriving maximum benefits from traditionally available therapeutic agents. Dosage optimization of existing medication regimens is one such method that can improve the efficacy of the anti-TB drugs.

The required drug concentrations of a highly effective TB therapy have already been reviewed and written into the guidelines by the WHO [2–4]. The most currently accepted norms for determining the dosage of anti-TB drugs are based on the recommendations made by WHOM [4]. Table 1 presents the summary of the recommended drug concentrations for TB treatment as given in the WHO guidelines. Though the regimens recommended based on these values are sometimes considered lengthy and complex, yet they are found to be highly effective in most of the cases [2]. However, the percentage of success of such anti-TB drug based therapies vary from country to country and is most often determined by the severity of the disease at the time of diagnosis. Furthermore, it has also been observed that the results of general practice often differ from the clinical trials. In addition, prevalence of low-concentration of anti-TB drugs in patients’ serum in clinical settings has been reported in many typical cases that are summarized in Table 2. Some of the most common conditions include unresponsiveness to anti-TB drugs, patients with difficult TB and existence of co-infection viz. HIV or diabetes mellitus. Moreover, it has also been observed that some pulmonary TB patients may still be under-dosed even after the administration of standard doses. This may be due to lack of customization of the dosages or variable drug pharmacokinetics. In such cases, administration of high-dose regimens of anti-TB drugs seems to be the most viable clinical choice.

**Table 1:** Summary of the WHO-recommended doses and the high doses recommended by clinical trials

Drugs	WHO-recommended dose				Recommended high dose	
	Daily		Three times per week		Daily dose	
	Dose and range (mg/kg body weight)	Maximum (mg)	Dose and range (mg/kg body weight)	Daily maximum (mg)	Dose and range (mg/kg body weight)	Maximum (mg)
Isoniazid	5 (4–6)	300	10 (8–12)	900	16–18	
Rifampicin	10 (8–12)	600	10 (8–12)	600		900–1200
Pyrazinamide	25 (20–30)	–	35 (30–40)	–		
Ethambutol	15 (15–20)	–	30 (25–35)	–	25	
Streptomycin	15 (12–18)	^a^	15 (12–18)	1000		
Kanamycin	15	1000	The same dose during the continuation phase			
Amikacin	15–20	1000	The same dose during the continuation phase			
Capreomycin	15–20	1000	The same dose during the continuation phase			
Ciprofloxacin	1000–1500		–			
Cycloserine E	10–15	1000	–			
Ethionamide	15–20	1000	–			
Gatifloxacin	400	–	–			
Levofloxacin	750	1000	–		17–20	1000
Moxifloxacin,	400	–	–			600
Ofloxacin	800	–	–			
*p*-Aminosalicylate	150	12,000	–			

Only the clinical trial results within combination regimens were shown in this review

^a^Patients aged over 60 years or weighing less than 50 kg may not tolerate 500–750 mg/day

**Table 2:** Low drug serum concentrations reported in different types of patients

Types of patients	Mechanisms	References
Common pulmonary TB with low-dose prescription	Some patients are underdosed even at standard doses	[75, 121–124]
	Fixed-dose combination with at least one low drug level in the serum	[14, 15, 82, 125]
Patients with slow response to TB treatment	Low serum level of Cmax 2 h post-dose	[126]
Patients with difficult TB	Difficult to increase the drug serum level	[106]
Patients with TB and HIV	Poor exposure to anti-TB drugs	[127, 128]
	Interaction between anti-HIV and anti-TB drugs	[129]
Patients with TB and diabetes mellitus	Decreased exposure to anti-TB drugs	[130]
	Differences in hepatic induction	[131]

Many clinical trials have been conducted to evaluate the clinical efficacy of administration of high dose anti-TB drugs and various guidelines and handbooks relevant to treatment and management of patients with MDR/XDR-TB (XDR-TB, defined as resistant to either INH or RFP, any fluoroquinolone, and at least one of three second-line anti-TB injectable drugs: capreomycin, kanamycin, and amikacin) have been updated and published based on the results obtained from these clinical trials [5–11]. Besides the injectable drugs the most commonly recommended high dosage medicine regimens for the treatment of RR-TB or MDR-TB includes levofloxacin (≥ 750 mg/day) in group A (fluoroquinolone, FQs) and INH in group D1 [Add-on agents including pyrazinamide (PZA), ethambutol (EMB) and high-dose INH] [11]. Although, high-dosage INH is the only higher dosage of anti-TB drugs recommended during the induction phase in the Bangladesh regimen, researchers are trying to formulate new and effective high dosage regimens [12].

Although, a great deal of progress has been made regarding the formulation and clinical implementation of high dose anti-TB drug regimens, the exact mechanisms underlying the effectiveness of such treatment methods is yet to elucidated. In the meanwhile, medical research has also made considerable progress in deciphering the molecular pathways involved in *Mycobacterium tuberculosis* (*M. tuberculosis*) and host interaction and clinical manifestation of the disease. This review summarizes the research revelations pertaining to host–pathogen interactions in TB that were made in the past decades. It also describes the bio-molecular and clinical aspects related to the need of administration of high-dose treatment strategies, discusses real time clinical results of the same and finally proposes some valuable suggestions that may contribute to accentuate the research and clinical implementation of high dose regimen for treatment of TB. The data presented here were collected from publications retrieved by searching in MEDLINE database. The keywords used for multiple searches included ‘high dose, tuberculosis, drug’. Other publications that were included were selected based on authors’ experience in the subject.

### Host variance

The hosts’ inherent attributes have a profound influence on both the pharmacokinetics and pharmacodynamics of the administered drugs, thus affecting their treatment outcomes. Factors that influence the therapeutic outcome of the regimen include—body weight, functioning of drug metabolic pathways, drug malabsorption, and the drug’s failure to reach the extra-pulmonary tissues (Table 3).

**Table 3:** Outline of possible explanations for anti-tuberculosis treatment failure

Types of reasons	Reason for treatment failure	Mechanisms	References
Host conditions	Body weight	Prescriptions without considering the body weight	[14, 15]
	Obesity	Impact on drug binding to albumin, increase in cytochrome P450 2E1 activity and phase II conjugation activity	[19]
	Special metabolism of the drug	Hepatic *N*-acetyltransferase 2 (NAT2) genotype affects the INH acetylator status and activity	[23, 27, 28]
	Malabsorption	Gut permeability and solubility; hepatic and renal clearance	[29, 30, 132]
	Failure to reach in EPTB	Anatomic barriers to drug penetration	[7, 133, 134]
Bacterial changes	Physical barrier of the cell wall	Increased dosage of anti-TB drugs might enhance drug permeation across the thicker cell wall into the bacilli	[40, 41]
		Formation of infectious biofilms	[43, 47, 48, 135]
	Drug efflux pumps	Efflux pumps are the first step in a general pathway to drug resistance	[58–61]
	Metabolic state of *M. tuberculosis*	Metabolic shutdown renders *M. tuberculosis* tolerant to a number of antibiotics	[62, 63]
	Special genotyping clinical isolates	Manu2 found to be significantly associated with mixed infections, resulting in hetero-resistance	[64, 65]

#### Body weight

The percentage of adults with body mass index (BMI) ≥ 25 kg/m^2^ on a global basis has increased from 28.8% in 1980 to 36.9% in 2013 among men, and from 29.8% in 1980 to 38.0% in 2013 among women. Accordingly, the number of people characterized as overweight and obese also increased from 857 million in 1980 to 2.1 billion in 2013 [13]. Analysis of the data of patients registered in Beijing Chest Hospital, China, between 1950s to 2010s, indicated that the average body weight has increased from 46.6 to 54.8 kg in women and from 55.2 to 61.5 kg for men. Though the recorded data point towards a change in the average weight and BMI of people across the world, the recommended dosage for anti-TB treatment regimens have not witnessed much modifications. Customized drug dosages based on body weight are seldom prescribed for individual TB patients. Instead, drugs are prescribed on a general basis as per the guidelines set by the WHO and the International Union against TB and Lung Disease fixed-dose combinations (FDCs). Furthermore, it has also been observed that these FDCs do not suffice the bioequivalence criteria.

As per the results of a comparative study conducted by Hao et al. [14] that analyzed the bioavailability of RIF and INH in patients administered with FDCs and single-drug formulations, the quantity of RIF in four out of the five FDCs was not within the required range. Furthermore, the INH quantity in one out of five FDCs was also found to fail in the bioequivalence criteria [14]. A randomized retrospective clinical trial conducted in Taiwan showed that about 20% TB patients received inadequate dosage of anti-TB drugs in three-drug or two-drug FDCs in the year 2003 [15]. Similarly, most physicians in China prescribe INH dose of 0.3 g/day without considering the patient’s body weight. Even though the WHO recommends to prescribe drug doses based on the body weight of the patient [16], proper dosing of anti-TB drugs remain to be highly inconsistent in many areas.

#### Obesity and nutritional status

It has been found that body composition, regional blood flow and capacity to bind with plasma proteins are the three main factors that affect the distribution of drugs in tissues and consequently their volume of distribution (Vd) [17, 18]. It is critical to consider the possible changes in the Vd of a drug when administered in an obese patient. Additionally, it is also important to consider that the altered pathophysiology of obese patients can influence both the processes of drug distribution and drug elimination [19]. However, no changes in drug absorption capacity of such patients have been observed yet [20–22].

Although pharmacokinetics of most drugs in obese patients are yet to be elucidated, it is highly recommended that in cases where such information is available, it should be implemented for designing modified treatment regimens that compensates for any significant differences in obesity induced plasma clearance and Vd of the patients.

Undernutrition status is one of the tuberculosis symptoms. Patients with TB combined with diabetes are the focus of the undernutrition population [23]. Evidence showed that male nutrition, especially the vitamins deficiency in A, D, and E, usually happened in tuberculosis patients [24] and the extension of time to negative sputum culture was related to BMI, white blood cell count (WBC), serum albumin and other non-nutritional factors [25].

#### Special drug metabolism

INH is one of the most popularly prescribed drugs whose therapeutic efficiency is affected by changes in the patients’ metabolism. INH is metabolized mainly by hepatic *N*-acetyltransferase 2 and cytochrome P450 2E1. The elimination phenotype and *N*-acetyltransferase 2 genotype are concordant, and three acetylator types can be distinguished: fast (homozygous FF), intermediate (heterozygous FS), and slow (homozygous SS) [26, 27]. A recent meta-analysis of 13 randomized studies, examined the influence of INH acetylator status on TB treatment outcomes [28]. Despite heterogeneity in companion drugs and dosing schedules of the studies that were included in the meta-analysis, it was found that fast acetylators had a twofold higher risk of microbiological failure and acquired drug resistance as compared to slow acetylators. Poorer microbiological outcomes in fast acetylators were observed, even in patients who were treated with three or more drugs. However, it was found that the risk of relapse was not significantly higher in fast acetylators than that in slow acetylators.

#### Drug malabsorption and clearance

Patients with renal failure warrant special attention in identifying and addressing drug malabsorption. In some patients who show early malabsorption; once the TB drugs begin to work the rates of absorption also improve. This was found to be particularly true for INH, with its concentrations rebounding later in treatment [19].

It is important to consider that all TB patients with renal failure are at high risk of accumulation of the drugs, especially EMB and streptomycin (SM), which may further cause adverse drug reactions in future. Certain metabolites, including pyrazinoic acid, 5-hydroxypyrazinoic acid, and acetyl-para-aminosalicylic acid also require renal clearance, while INH, RIF, PZA, ethionamide (ETH), and *p*-aminosalicylic acid (PAS) are predominantly cleared hepatically [29, 30]. Therefore, patients with significant renal or hepatic dysfunction require prior assessment of serum concentrations so that the most appropriate drug dosage is calculated and prescribed.

Apart from drug interactions, the presence of other diseases may also affect the absorption of the drugs. The same has been observed in HIV +ve TB patients with various forms of enteropathy [31–33]. This may be particularly problematic in the case of RIF, for which absorption is dependent upon gut permeability and solubility, with the latter being affected by both pH and gut transit time [34].

#### Failure to reach extra-pulmonary tissue

While the treatment of most forms of EPTB does not differ significantly from that of pulmonary TB, meningitis and bone/joint disease, the treatment regimens recommended by WHO guidelines may however require longer treatment durations [4]. Furthermore, considering the important roles of anatomic barriers in drug penetration, the study of treatment of EPTB may help in studying dosage optimization. Although, administration of higher doses of RIF for the treatment of TB meningitis have not yet been formally recommended, a recent open-label, computer-based medicine-assignation random clinical study that was conducted in Indonesia revealed the presence of lower RIF concentrations in cerebrospinal fluid than in plasma. Furthermore, it was also found that patients treated with higher doses of intravenous RIF during the first 2 weeks of therapy exhibited only 35% 6-month mortality, which is significantly lower than the recorded 65% in patients treated with standard-dose RIF [6]. The increased dosages of RIF were not associated with the incidence of any adverse effects.

Hence, it can be concluded that the factors that must be considered while formulating more efficient TB treatment regimens are multi-factorial. The medical condition of the patient is one of the most crucial factors that the clinician must consider while prescribing the anti-TB drugs. The high dose regimens are typically administered with the aim to improve the rapidity of the clinical response, while being careful of limiting the possible adverse effects they may inflict.

The timely adjustment of drug therapy based on the above listed host variance indicated the requirement for the therapeutic drug monitoring (TDM). The use of TDM is not yet standard in the treatment of TB and no official guidelines for TDM are available at international (e.g. WHO) or national health organizations [8]. When multiple blood samples in the clinical setting are impossible the most frequent used sampling time points are recommended at 2 and 6 h post-drug intake. The first sample for the 2-h post-dose concentrations can reflect the peak plasma concentration, but can’t distinguish between delayed absorption (late peak, close to normal range) and malabsorption (low concentrations at all time points). The second sample, often collected at 6-h post-dose, can differentiate between these two scenarios and also provide some information about clearance and half-life, assuming that drug absorption was nearly completed by 2 h [35]. Dried blood spot analysis and limited sampling strategies might provide us with a more patient friendly approach [8].

## Bacterial changes

Ever since the anti-TB drugs were developed they have been used continuously across the world without any significant changes. In the meanwhile, the *M. tuberculosis* bacilli have also been going through several structural and molecular changes. These changes are manifested for human beings as the increasing appearance of multidrug-resistant TB. High-dosage treatment regimens may be effective in treating infections in which the bacteria has changes in the physical barriers, efflux pump, as well as genetic constitution.

### Cell wall as the physical barrier

The cell wall of *M. tuberculosis* consists of complex lipids. It also includes the significant peptidoglycan–arabinogalactan–mycolic acid wall structure, which acts as a permeability barrier against the penetration of chemical drugs [36–38]. This outer layer of *M. tuberculosis* changes with the stimulation of anti-TB drugs, becoming rougher and thicker. A thorough atomic force microscopic analysis of the bacteria that was conducted by Alsteens et al. demonstrated that INH, RIF, EMB and SM can induce a substantial increase in surface roughness of *Mycobacterium bovis* (*M. bovis*) BCG [39]. The results obtained by Velayati et al. further confirmed that the increase in surface roughness of the XDR-TB bacilli is even more prominent as compared to the susceptible bacilli [40]. Altogether, these results indicated that the therapeutic efficiency of a drug may change progressively with the erosion of the envelope—and evidently not for the better. Comparisons of the cell walls of XDR-TB, MDR-TB, and susceptible TB bacilli showed marked differences in the thickness of the cell walls (p < 0.05) under Transmission Electron Microscope. The XDR-TB bacilli were found to have the thickest cell wall and most dense basal peptidoglycan layer [41]. Thus, it is possible that increase in dosage of anti-TB drugs can enhance drug permeation into the bacilli.

### Biofilm formation

Biofilms are surface-associated multicellular communities that develop when bacteria adhere to extracellular polymeric substances [42]. Many bacteria including *M. tuberculosis* can develop biofilms [43]. Regardless of the causative pathogen, infectious biofilms demonstrate extraordinary tolerance to antibiotics and subversion of the host’s immune system [44–46]. Preliminary evidence suggests that biofilm formation is one of the main factors that may contribute to the pathogen’s persistence to antibiotics in in vivo survival of *M. tuberculosis* [47, 48]. Although no clearly direct evidence showed that exposure to treatment could induce biofilm formation, our studies on the biofilm formability by crystal violet staining absorption method showed that the OD value of retreatment group was higher than that of the initial treatment group (*p* = 0.016) and the biofilm formation is positive related with the number of resistant drugs (*r* = 0.185, *p* = 0.002) [49]. The ability of *M. tuberculosis* to form biofilms may promote its multiplication and persistence [50]. Thus, the factors that disrupt biofilm structure or affect biofilm formation are regarded as efficient methods of inhibiting biofilm growth, disrupting already formed biofilms and hence combating the infection. Taraszkiewicz et al. summarized such factors for different microorganisms that contribute to the formation of biofilm viz. enzymes, sodium salts, metal nanoparticles, antibiotics, acids, chitosan derivatives and plant extracts [51]. Unfortunately, not many effective anti-TB drugs that target and inhibit the formation of *M. tuberculosis* biofilms are discovered yet [43].

### Efflux pump

Bacterial drug efflux pumps are classified into five families: the ATP-binding cassette superfamily [52], the major facilitator superfamily [53], the multidrug and toxic compound extrusion family [54], the small multidrug resistance family (a subgroup of the drug/metabolite transporter superfamily) [55], and the resistance-nodulation-division superfamily [56–58]. Research studies have proved that *M. tuberculosis* bacteria contain at least two or three dozen putative drug efflux transporters [58, 59]. The induction of these efflux pumps is considered the first step towards the development of drug resistance that eventually leads to the development of high-level, chromosomal mutation-related resistance in these bacteria [60]. Research data on the activity of these efflux pumps against FQs indicate that increasing ofloxacin MICs are responsible for increased activity of the FQ efflux pump [61]. High doses of anti-TB drugs can overcome the effect of such efflux pumps and kill the infecting bacilli more efficiently.

### Metabolic state of *M. tuberculosis*

The susceptibility of *M. tuberculosis* to specific drugs is influenced by a variety of metabolic stresses, such as the presence of antibiotics, hypoxia, and low pH in the macrophage lysosome. The resulting metabolic shutdown renders *M. tuberculosis* tolerant to many antibiotics, suggesting a direct link between bacterial metabolic state and observed drug efficacy [62]. The study conducted by de Steenwinkel et al. investigated the time-kill kinetics of anti-TB drugs in relation to the metabolic activity of *M. tuberculosis* and found that when compared with highly active mycobacteria elimination of the susceptible low-activity mycobacteria requires 64-fold increase in INH concentration and a fourfold increase in RIF concentration, whereas amikacin (AMK) was equally effective irrespective of the mycobacteria’s metabolic state [63]. These results indicate that the metabolic state of *M. tuberculosis* bacteria significantly affect its susceptibility to antimicrobials, and hence exert a profound effect on the optimization of anti-TB drug dosages required to maximize the reduction in *M. tuberculosis* load and minimize the emergence of drug resistance.

### Special genotyping isolates

Manu2, one of the genotypes sorted by spoligotype number, was found to be significantly associated with mixed infections among the 499 tested clinical isolates (odds ratio 47.72; confidence interval 9.68–235.23; *p* < 0.01). Four isolates (1.37%) were confirmed to be hetero-resistant, out of which three (75%) were caused by mixed infections and belonged to Manu2 [64]. That study first revealed that Manu2 was the most predominant genotype in cases of mixed infections and potentially the main reason behind development of hetero-resistance. The results obtained from the study conducted by Mei et al. further confirmed the presence of correlations between mixed infections and Manu2. The MICs of Manu2 were low, suggesting that increased treatment dosages may be able to kill the special Manu2 genotypes [65].

### Possible role of next generation sequencing in identifying bacterial changes during high-dose therapy

Whole-genome sequencing and next-generation sequencing data can be used for directing the process of development during the high-dose therapy. It can also be used to detect and understand the process of microevolution within the *M. tuberculosis* lineages [66] or populations [67], and comprehend the epidemiology [68–70], and mutation rate [68, 69, 71] of the same. Since reversion mutations are rare [72], it is relatively easy to observe and locate the mutations in *M. tuberculosis* populations at various stages of the disease.

Thus, it can be concluded that during the past decades, *M. tuberculosis* bacteria have developed thick cell wall, high efflux pump activity and even adopted to use their special metabolic pathways to resist the activity of the drugs. These effects are considered to be the indirect outcomes of continuous use of anti-TB drugs. Administration of high dosage of the anti-TB drugs may serve to partially overcome this resistance in the bacteria, but conquering the fight against TB necessitates the development of advanced versions of routinely used antibiotics and the simultaneous development of more effective brand-new ones.

#### Clinical outcomes of high dose treatments

High-dosage TB treatment was first tried with RIF in the observational comparative clinical studies more than four decades ago [73]. More recent approaches of treating drug-resistant TB employ high doses of anti-TB drugs, especially RIF [18, 74], INH [75] and moxifloxacin [76]. Detailed weight-based drug dosing for adults can be found in Annex 2 of the WHO guidelines for treating TB [4, 77]. To add to the existing levels of understanding of mode of operation of these high-dose treatment regimens Dooley et al. recommended that the topic should be considered as a research priority and studied extensively [5]. In the following discussion, the clinical results obtained by the administration of combination regimens for treating TB that usually lasts at least 1 or 2 months are discussed, rather than emphasizing early bactericidal activity.

*INH* INH has been used as one of the first-line drugs for the treatment of TB for nearly 60 years. The recommended dose of INH for daily regimens is 5 mg/kg (range 4–6 mg/kg), with a maximum dose of 300 mg, as recommended by WHO [4]. It may also be used in thrice-weekly regimens at 10 mg/kg (range 8–12 mg/kg), with a maximum dose of 900 mg [4]. Genotype-based dose individualization has been suggested for INH in a random clinical study. It was also proposed that to achieve uniform INH exposure, doses of 2.5, 5, and 7.5 mg/kg are appropriate for the treatment of homozygous slow, heterozygous fast, and homozygous fast acetylators, respectively [78].

The *inh*A gene is part of the FAS-II fatty acid elongation system required for mycolic acid synthesis [6, 79]. Mutations in the *inh*A promoter region are a less common cause of INH resistance than the mutation of the *katG* gene. However, it is important to recognize the strains with this mutation because they are highly resistant to the thioamides [6, 80]. The INH resistance caused by mutations of the *inh*A promoter region can be overcome with higher dosing of INH, around 10–15 mg/kg/day [81].

According to the results of a randomized clinical trial, individuals with MDR-TB and receiving high doses (16–18 mg/kg) of INH tested sputum-negative 2.38 times (95% CI 1.45–3.91, p = 0.001) more rapidly and were 2.37 times (95% CI 1.46–3.84, p < 0.001) more likely to be sputum-negative after 6 months than those not receiving the high doses [82]. Furthermore, the high-dose administered patients also show significantly better radiological improvement without inducing any toxic responses [82]. In another randomized controlled study, an INH dose of 10 mg/kg/day demonstrated bactericidal activity against an *inhA* promoter mutant, and 25 mg/kg/day had a bactericidal effect against a *katG* mutant (AUC/MIC of approximately 40 and 15, respectively) [83]. It is thus proposed that higher INH doses have bactericidal effects against strains with MICs of 1 or 2 μg/mL, but the final clinical outcome is dependent on the acetylator status of the patient. However, it was also observed that peripheral neuropathy was a common outcome of high-dose INH treatment. Therefore, it may be concluded that administration of high doses of INH may be useful in the treatment of drug-resistant TB, but its clinical efficacy will depend on the dose, patient acetylator status, and degree of INH resistance.

*RIF* Optimizing the dosage of RIF and its cyclopentyl derivative, rifapentine, holds the greatest promise for ensuring better treatment outcomes for current first-line treatment methods that need to be followed for more than 6 months, especially in patients who harbor fully drug susceptible TB [72, 84]. The results of a randomized clinical trial that were presented by van Crevel et al. demonstrated that although treatment with 10 mg/kg of RIF failed to produce the reference concentration of greater than 8 mg/L, higher doses were significantly correlated with peak plasma concentrations above the reference value [85]. In South Africa, Diacon et al. demonstrated that the early bactericidal activity was almost doubled upon the administration of twice the standard dose of RIF in patients with smear-positive pulmonary TB [86]. In Indonesia, an open-label phase II randomized clinical trial administered standard (450 mg) or high (600 mg) doses of RIF in 46 individuals, 23 in each treatment group. The results showed that mean plasma concentrations within each treatment group were similar during the weeks 4 and 8. In week 4, the percentages of patients with RIF peak plasma concentrations above 8 mg/L among the 450-mg dose group was 48%, while the 600-mg dose group was 78% (χ2; p = 0.03) [87]. To further evaluate the efficacy and safety of higher RIF doses for the treatment of pulmonary TB, Steingart et al. performed a systematic review of the existing data and concluded that patients receiving at least 900 mg RIF had higher chances of culture conversion. At the same time an increase in the incidence of flu-like symptoms was also observed [88]. The summarized results of multiple studies on TDM showed that the administration of increased doses of RIF result in adequate peak concentrations in plasma and improved treatment results [16]. In an early report on RIF, administration, increasing the dosage from 10 to 20 mg/kg daily resulted in fourfold increase in the AUC0-24 and enhanced the early bactericidal activity required for the treatment of naïve pulmonary tuberculosis patients [89]. Furthermore, the randomized clinical trial also showed that increasing the dosage of RIF (10, 15, or 20 mg/kg) also shows better culture negative rates in solid and liquid medium without increasing the percentages of the treatment-discontinuing participants [90]. Phase II clinical trials with the same dosage of RIF showed that the maximal treatment efficacy of the drug is likely to be achieved at 1200 mg/day. However, patients with large lung cavities were observed to be less responsive to the treatment [91]. Jindani et al. reported that for those patients with newly diagnosed, smear-positive, drug-sensitive tuberculosis, the 6-month regimen that included weekly administration of high-dose of RIF (900, 1200 mg) and moxifloxacin was as effective as the control regimen recommended by WHOM [92].

It was also observed that administration of high-dose RIF can also shorten the duration of therapy, perhaps to 4 months or less. An early observational comparative study indicated that delivery of 1200 mg RIF daily can achieve a nearly-complete negative sputum culture results after only 90 days, without exerting any additional adverse effects [93]. On the other hand, it was found that approximately 35% of the patients who received 450, 600, or 750 mg of RIF within daily combination therapy had converted within 1 month, while almost 70% had converted by the end of the next month. While, only 60% of those receiving 450 mg and 75% of those receiving 750 mg of RIF had converted by the end of the 2nd month [94]. When the outcomes of administration of the 600 mg/day dose was compared with that of 1200 mg/day it was found that the later regimen had high frequency of culture conversion in humans in the 1st and 2nd months which was found to be consistent with the results obtained from mouse model studies [94, 95]. Recent observational comparative study tried four treatment regimens (35 mg/kg/day RIF + 15–20 mg/kg/day EMB, 20 mg/kg/day RI F + 400 mg moxifloxacin, 20 mg/kg/day RIF + 300 mg SQ109 and 10 mg/kg/day RIF + 300 mg SQ109) and the daily standard control regimen (10 mg/kg RIF, 5 mg/kg INH, 25 mg/kg PZA and 15–20 mg/kg EMB). The results showed that the high dose of 35 mg/kg RIF was safe and it could help in reducing the time of culture conversion in liquid media; and can thus be considered as a promising component of future, shorter treatment regimens [96]. SQ109 is a well-tolerated drug candidate based on the ethylene diamine pharmacophore [97].

Summing-up, research studies and clinical trial results have clearly demonstrated that the high-dose RIF treatment regimens significantly improve the sterilizing activities as compared to the standard doses, without increasing the chances of occurrence of any adverse events [98–100]. Thus, application of high-dose RIF treatment regimens represent one of the most direct methods of improving the outcomes of the existing first-line drugs and shortening the duration of TB treatment. Conduct of even more number of clinical trials in countries with varying TB burdens can contribute to either validating or negating this hypothesis.

*FQ* Among the extant anti-TB agents, FQs constitute the most crucial class of drugs that are known for treating MDR-TB without cross-resistance. They are known to achieve better clinical treatment outcomes than all other commonly administered drug groups [6, 101]. The FQs operate by inhibiting the function of *M. tuberculosis*’s DNA gyrase and obstructing replication and transcription [102]. The most common and effective types of FQs include ciprofloxacin, ofloxacin, levofloxacin, moxifloxacin, and gatifloxacin.

Early in 1990, Yew et al. investigated the dose-dependent effectiveness of FQs in treating MDR-TB and the results of these observational comparative studies showed that patients administered with ofloxacin (800 mg) once daily had more rapid sputum culture conversion efficiency than those administered with 300 mg once daily of the same drug [8]. The earlier proposition was challenged when Chigutsa et al. suggested that the recommended ofloxacin dose (800 mg) is inadequate for the treatment of majority of the patients with pulmonary tuberculosis [103]. The probability of target attainment population in the study was 0.45. However, doubling the dose to 1600 mg increased it to 0.77 [103]. Similarly, the results of target attainment analysis done by Alsultan et al. suggested that the efficacy of levofloxacin can be improved by using higher doses (17–20 mg/kg of body weight) of the drug [104].

Furthermore, HIV infection was not found to have any significant effects on the ofloxacin pharmacokinetics [105]. However, previous reports on possible side effects of FQs, including the occurrence of dysglycemia, tendonitis, anemia and Q–T interval prolongation necessitates further evaluation of the safety issues associated with administration of higher ofloxacin doses [106]. Another observational comparative study showed that short-term moxifloxacin treatment (600 mg/day) for 6 months is equally effective as that of treatment with 400 mg/day for 9 months. However, the probabilities of adverse effects and drug resistance decrease significantly in short-term treatment regimens [74]. An open-label randomized controlled Phase II trial that tested the therapeutic efficacy of administration of 800 mg moxifloxacin once daily for treating TB meningitis revealed a proportional increase in plasma AUC(0–6), C(max) and drug concentrations in the cerebrospinal fluid and good tolerance when administered for 14 days [7]. The International Union Against TB and Lung Disease, in partnership with other institutions and agencies, conducted the STREAM (Evaluation of a Standardized Treatment Regimen of Anti-tuberculosis Drugs for Patients with Multidrug-resistant Tuberculosis) trial in multiple locations from across the world and evaluated the efficacy and safety of high-dose moxifloxacin (600–800 mg) administered once daily in a standardized regimen for the treatment of MDR-TB [107]. Levofloxacin at a high dose of 1000 mg/day was found to produce maximum plasma concentration, largest volume of distribution, and longest elimination half-life in comparison with gatifloxacin or moxifloxacin (400 mg daily) [108]. The study conducted by Johnson et al. observed that levofloxacin (1000 mg/day) had the similar early bactericidal activity as that of INH, and better than that of moxifloxacin (400 mg/day) and gatifloxacin (400 mg/day) [109]. Considering the cost and effectiveness of FQs, high-dose levofloxacin was recommended in the WHO MDR-TB treatment guidelines [101]. The article by Yew and Nuermberger summarized the previous data on clinical application of FQs and concluded that their high dose administration is an efficient method to shorten the total duration of therapy [110].

*EMB* In the 1970s, Radenbach remarked that doses of EMB below 15 mg/kg cannot prevent the emergence of resistance to companion drugs. Therefore, 15 mg/kg/day is considered the clinical minimal effective dose for EMB [111]. When administered in combination with INH, EMB (25 mg/kg) shows superior 6-month culture conversion rates, as compared to 12.5 mg/kg. It is thus proposed that EMB is highly active at a daily dosage of 25 mg/kg in combination with a good sterilizing agent [112]. On the flip side, EMB-related optic neuritis is known to be dose- and duration dependent and the effects may sometimes be irreversible. Higher daily doses like 25 mg/kg are probably more active, but they also increased the risk of ocular toxicity [76]. The incidence of this complication is around 5% at the 25 mg/kg/day dose, and less than 1% at the 15 mg/kg/day dose. Some other studies suggest that the risk may be decreased by following the thrice-weekly dosing [113].

*PZA* The recommended dose for PZA in adult patients is 25 mg/kg (range 20–30 mg/kg) and 35 mg/kg (range 30–40 mg/kg) for daily and thrice-weekly dosing, respectively [12]. The standard daily dose is usually rounded to 2 g/day in patients weighing more than 50 kg, and 1.5 g/day in those weighing less than 50 kg.

It has been shown that doubling the human-equivalent PZA dose increases bactericidal and sterilizing effects in mice and guinea pigs [114]. Importantly, PZA also shows synergistic effects when administered together with investigational TB drugs.

In summary, high dose treatment regimens were found to shorten the therapy duration for MDR-TB, especially the RIF and FQs. The same effects were also observed when INH was used in the treatment of XDR-TB, which according to many specialists should be individualized based on the contact history, drug history etc. Moreover, the clinical manifestation of tuberculosis was not restricted to lungs; rather it was known to infect other organs and tissues, most of which were hard to be penetrated by the conventional anti-TB drugs, thus, causing low drug concentration at the lesion. Clinical trials seldom reported success of high-dose treatment of extra pulmonary tuberculosis, except for RIF and INH [7, 115]. It could thus be concluded that whenever the clinical conditions of a patient demanded the administration of high dose treatments, it is highly important to evaluate the possible toxic responses viz. hepatotoxicity and flu-like syndrome; it may elicit [72].

## Conclusions

This review summarized the possible reasons why the plasma serum concentrations of anti-TB drugs can’t meet the clearance of the pathogen. However, this should not be considered that the current strategies of treatment of TB are completely ineffective. In fact, the standard regimens cure around 90–98% of patients [35]. Furthermore, a majority of patients who receive a total of 6 months of treatment for drug-susceptible TB recover completely with no future complications or recurrence [116, 117]. For the XDR or pre-XDR TB six or seven drug regime were recommended, which included four new drugs (linezolid, bedaquiline, clofazimine/cycloserine and carbapenem/delamanid) plus two or three possible supporting drugs and only the supporting drugs showed the evidence of the high dosage [118].

Although many studies have demonstrated that application of higher doses of anti-TB drugs is a highly promising treatment strategy, there are several limitations associated with it that still remains to be addressed. One of the most important issues is accessing and preventing the adverse effects of high-dose anti-TB drugs. Furthermore, high doses of antibiotics may also trigger strong selections of pathways responsible for drug resistance development in *M. tuberculosis*. The selection strength can influence the complexity of the antibiotic resistance developed. The use of high doses of antibiotics to clear infections may also promote the increase the number of cases of cross-resistance in clinical settings [119, 120]. As of yet, there is insufficient data on direct, head-to-head comparison of the safety and overall therapeutic outcomes of daily administration of the current standard doses of drugs against the daily administration of higher doses.

In conclusion, low concentrations of anti-TB drugs should be dealt with extreme caution as it may influence the pathogen’s drug susceptibility and the short-course treatment strategy for fighting TB. A better and easier way to overcome low serum concentrations of anti-TB drugs is to prescribe high doses for TB treatment, subject to prior verification of absence of adverse effects and cross drug resistance. Taking also into account the increase in adverse effects and the host/bacterial factors, It is becoming a critical point to identify if those patients in whom high-dose treatments are truly cost-effective.

## Abbreviations

TB: tuberculosis
WHO: World Health Organization
MDR-TB: multidrug-resistant TB
RR-TB: rifampicin-resistant TB
FDCs: fixed-dose combinations
RIF: rifampin
INH: isoniazid
Vd: volume of distribution
EMB: ethambutol
SM: streptomycin
PZA: pyrazinamide
PAS: *p*-aminosalicylic acid
TDM: therapeutic drug monitoring
EPTB: extra-pulmonary tuberculosis
BCG: Bacillus Calmette–Guerin
XDR-TB: extensively drug-resistant tuberculosis
FQ: fluoroquinolone
MIC: minimal inhibitory concentration
AM: amikacin
AUC: area under curve
STREAM: evaluation of a standardized treatment regimen of anti-tuberculosis drugs for patients with multidrug-resistant tuberculosis
